# Dielectric Spectroscopy of PP/MWCNT Nanocomposites: Relationship with Crystalline Structure and Injection Molding Condition

**DOI:** 10.3390/nano11020550

**Published:** 2021-02-22

**Authors:** Marco Monti, Marta Zaccone, Alberto Frache, Luigi Torre, Ilaria Armentano

**Affiliations:** 1 Proplast, Via Roberto di Ferro 86, 15122 Alessandria, Italy; marta.zaccone@proplast.it; 2 Department of Applied Science and Technology, Polytechnic of Turin, INSTM Research Unit, Viale Teresa Michel 5, 15121 Alessandria, Italy; alberto.frache@polito.it; 3 Civil and Environmental Engineering Department, University of Perugia, Strada di Pentima 4, 05100 Terni, Italy; luigi.torre@unipg.it; 4 Department of Economics, Engineering, Society and Business Organization (DEIM), Tuscia University, Largo dell’Università snc, 01100 Viterbo, Italy

**Keywords:** carbon nanotubes, polymer nanocomposites, dielectric properties, crystallinity

## Abstract

In this paper, we study the correlation between the dielectric behavior of polypropylene/multi-walled carbon nanotube (PP/MWCNT) nanocomposites and the morphology with regard to the crystalline structure, nanofiller dispersion and injection molding conditions. As a result, in the range of the percolation threshold the dielectric behavior shifts to a more frequency-independent behavior, as the mold temperature increases. Moreover, the position further from the gate appears as the most conductive. This effect has been associated to a modification of the morphology of the MWCNT clusters induced by both the flow of the molten polymer during the processing phase and the variation of the crystalline structure, which is increasingly constituted by γ-phase as the mold temperature increases. The obtained results allow one to understand the effect of tuning the processing condition in the frequency-dependent electrical behavior of PP/MWCNT injection-molded nanocomposites, which can be successfully exploited for an advanced process/product design.

## 1. Introduction

In the last decades, scientists all over the world have paid attention to carbon nanotubes (CNTs), because of their outstanding electrical properties [1]. Moreover, thanks to their tubular shape with a very high aspect ratio, they can turn the insulating behavior of polymers into conductive, even at very low content [2]. This is particularly interesting because a low CNT fraction does not modify the mechanical performance of the pristine polymer in which they are embedded, differently from other carbon-based additives (e.g., carbon black or graphite), which usually lead to brittle materials, due to the considerable amount needed to significantly modify the electrical behavior [3,4,5,6,7,8].

It is widely known that the processing technique can influence the morphology of a polymer nanocomposite [9]. As for the injection molding of thermoplastic polymers, fibrous and lamellar fillers are typically oriented in the flux direction [10,11]. This was also demonstrated for CNT-based nanocomposites [12]. Several studies have shown that the variation of the processing condition of injection-molded components, manufactured with CNT-based nanocomposites, can lead to drastic modification of the electrical conductivity, especially when the CNT content is in the range of the electrical percolation [13,14,15,16,17,18]. Villmow et al. [16] studied injection-molded polycarbonate-based CNT nanocomposites, demonstrating that lowering the cooling rate and residence time seems to favor the attainment of higher electrical conductivity. Several researchers focused their attention to semi-crystalline polymers [17,18]. For instance, Ameli et al. [17] studied injection-molded PP/CNT nanocomposite foams, finding that an increase of the injection flow rate favored the electrical conductivity. The relationship between processing condition and resulting crystalline structure was studied by Von Baeckmann et al. [19], who found the PP γ-phase formation is favored with a reduction of the cooling rate. However, to the best of our knowledge, very few efforts have been conducted in order to correlate the crystalline structure of nanocomposites with electrically conductive nanofillers and their resulting electrical features [20,21,22,23]. In a previous paper by the same authors [24], the effect of the injection molding condition on the DC electric properties of PP/multi-walled carbon nanotube (MWCNT) nanocomposites was investigated, showing that rising up the temperature of the mold and the injection rate, a decrease of electric resistivity can be attained. This was connected to the formation of a crystalline structure based on a relevant fraction of γ-phase, and the resulting inter-cluster morphology of the conductive network based on MWCNT.

Dielectric spectroscopy allows the study of the frequency-dependent AC electrical properties. It can be used as a valuable support for correlating the morphology of a polymer nanocomposite filled with conductive additives, with its electrical behavior, which may be masked under the DC approach [25,26,27,28,29].

In this paper, we report a study on the dielectric behavior of multi-walled carbon nanotubes (MWCNTs)/PP nanocomposites. As a result, the AC dielectric properties and the related electrical relaxations have been correlated to the morphology of the MWCNT clusters and the crystalline structure of PP, emphasizing the effect of the variation of the processing condition on the other characteristics. In particular, the study has highlighted the effect of rising the temperature of the mold for increasing the electrical conductivity and the mobility of the electric dipoles attributed to the polymer/tube interface, allowing the correlation with specific crystalline structures and MWCNT cluster morphology.

## 2. Materials and Methods

### 2.1. Materials

An injection molding grade PP (Moplen RP348R), produced by LyondellBasell (Rotterdam, The Netherlands), was selected as a matrix. As claimed by the manufacturer, it is a random copolymer, with a melt flow index equal to 25 g/10 min (230 °C, 2.16 kg). Multi-walled carbon nanotubes (NC7000) were purchased from Nanocyl (Sambreville, Belgium). As reported by the manufacturer, a catalytic carbon vapor deposition process was used for their production. Their average diameter was 10 nm and they were 1.5 µm long, with a surface area of 250–300 m^2^/g. The fraction of pure carbon in their composition is 90%.

### 2.2. Processing

Several MWCNT contents were selected to be added to PP (1, 2, 3, 4, 5, 6, 7 wt%), which have been homogeneously mixed by a co-rotating twin-screw extruder, Leistritz 27E (Nuremberg, Germany). The diameter (D) of the screw is 27 mm, with a length of 40D. A constant screw speed of 220 rpm was fixed. The temperature profile was set in the range of 190–200 °C. MWCNT have been added through a gravimetric dosing unit (Brabender FlexWall, Duisburg, Germany). An injection molding machine, Ferromatik K-Tec 200 with a diameter D of the screw of 50 mm, was used to process the nanocomposites. Samples with a rectangular shape (100 × 140 × 2 mm^3^ in size) were prepared. The temperature of the mold was fixed at 25 °C and a flow rate of 70 cm^3^/s was used. These parameters were referred to as standard conditions hereinafter.

Samples were also produced with two different mold temperatures (70 °C and 100 °C), in order to study its effect on the morphology and on the dielectric behavior of the nanocomposites. A temperature of the mold equal to 100 °C is typically considered very high for a polymeric matrix like PP and it has been obtained through the so-called Heat&Cool (H&C) process. A Vario-Therm control unit (HB-Therm, St. Gallen, Switzerland), kindly supplied by Nickerson Italia, was used to obtain this process. It consists of a dynamic heating of the surface of the mold, which is heated to high temperature during the injection phase, followed by a rapid cooling during the packing phase. This processing parameter was tuned for the nanocomposites containing 3 and 4 wt% MWCNTs. Throughout the paper, samples will be mentioned as “Xwt%-MWCNT T-Y°C”, with X indicating the MWCNT content and Y the used mold temperature.

### 2.3. Characterization

**Figure 1:**
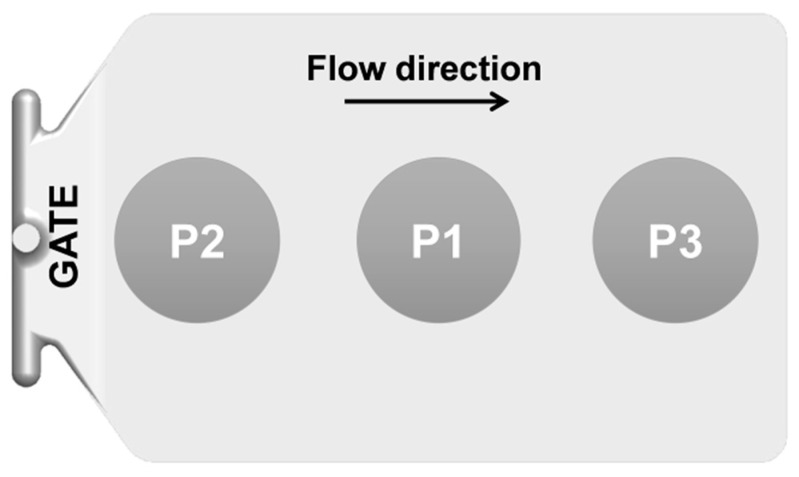
Schematic sketch of the sample and the related studied positions.

AC dielectric characterization was performed in the 10^1^–10^6^ Hz frequency range, using a HP 4284A precision LCR meter (Agilent, Santa Clara, USA). The used test fixture has an electrode surface area of 4.90 cm^2^. All the tests have been performed by applying a voltage amplitude of 0.5 V at room temperature. The samples were positioned between two copper-plated electrodes and the complex impedance $Z^{*}=Z^{\prime }-iZ^{\prime \prime }=\left|Z\right|e^{-i\theta }$ was obtained by measuring real part (*Z*’), imaginary part (*Z*”), module $\left|Z\right|=\sqrt{{Z^{\prime }}^{2}+{Z^{\prime \prime }}^{2}}$ and phase angle (*θ*) of impedance with respect to the frequency. Starting from these measurements, the bulk conductivity *σ_AC_* was computed according to the equation $\sigma_{AC}=\frac{1}{\left|Z\right|}\cdot \frac{d}{A}$, where *A* is the area of contact and *d* is the thickness of the specimen. The complex permittivity $\varepsilon^{*}=\varepsilon^{\prime }-i\varepsilon^{\prime \prime }$ was calculated from the equation $\varepsilon^{*}=\frac{1}{Z^{*}i\omega \;C_{0}}$, where $C_{0}=\varepsilon_{0}\frac{A}{d}$ is the vacuum capacitance and *ε*_0_ is the vacuum permittivity (8.854 × 10^−12^ F/m). Based on the impedance values, the real (*ε*’) and imaginary (*ε*’’) parts of complex permittivity can be calculated as $\varepsilon^{\prime }=\frac{Z^{\prime \prime }}{{\left|Z\right|}^{2}\omega C_{0}}$ and $\varepsilon^{\prime \prime }=\frac{Z^{\prime }}{{\left|Z\right|}^{2}\omega C_{0}}$. Tests were performed in three different positions over the surface of the samples, according to the scheme reported in Figure 1. Position 1, hereinafter referred to as P1, is the center position of the sample: all the results reported in this paper, are based on experimental tests in this position, wherever not specified differently. Position 2, hereinafter referred to as P2, is the position closest to the gate, which represents the part firstly reached by the molten material. Position 3, hereinafter referred to as P3, is the position furthest from the gate.

The morphology of the obtained nanocomposites has been studied according to two different approaches: one focused on the crystalline structure, the other to the morphology of the MWCNT clusters embedded in the polymer matrix. X-ray diffraction (XRD) and DSC were used to study the crystalline structure of PP. XRD analysis was performed using a Panalytical X’Pert PRO (Cu Kα radiation, wavelength of 1.54187 Å, Malvern Panalytical, Malvern, UK) diffractometer, from 2 to 30° 2*θ* with a step rate of 0.026°/min. Differential scanning calorimetry (DSC) was performed on a representative specimen cut from the whole cross-sectional area of the injection-molded sample. A Q800 equipment, from TA Instruments, was used. A single heating scan was set from 25 to 190 °C, with a heating rate of 10 °C/min. Field emission scanning electron microscopy (FESEM) was used to investigate the morphology of the MWCNT clusters in the cross sectional area taken in different position over the surface of the samples. Micrographs were taken by means of a Zeiss Merlin 4248 FESEM instrument (Carl Zeiss, Oberkochen, Germany). The specimens were cryo-fractured in liquid nitrogen and coated with a thin layer (<10 nm) of chromium before observation, using a Sputter Coater BAL-TEC SCD (Balzers, Liechtenstein).

## 3. Results and Discussion

### 3.1. Dielectric Analysis

#### 3.1.1. Effect of the MWCNT Content 

**Figure 2:**
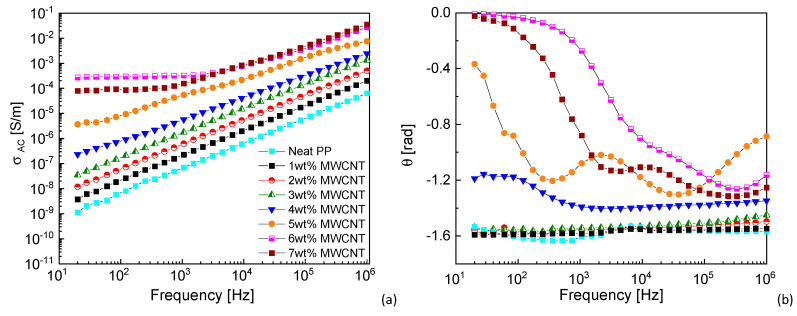
(**a**) AC electrical conductivity and (**b**) phase angle of the neat PP and the studied nanocomposites (tests performed in P1; graph legend reported in (**a**) is valid also for (**b**)).

Dielectric properties were studied as a function of MWCNT content and the results are reported in Figure 2. Figure 2a shows the electrical conductivity (*σ_AC_*) as a function of frequency (log-log plot). The electrical conductivity of neat PP increases with frequency in the whole frequency range, as expected for insulating materials [29]. The same behavior can be observed for MWCNT nanocomposites with the lower nanofiller contents. Indeed, when the concentration of MWCNTs is lower than the percolation threshold, the nanocomposite acts as an insulator and its electrical conductivity is frequency-dependent because of the hopping and tunneling mechanism [30]. As the frequency increases, the capacitive part, which can be associated to both the capacitive behavior of the polymer matrix and to the contribution of the tube/polymer/tube structures, contributes to an increasing conductance directly proportional to the frequency (*iωC*).

When the concentration of MWCNTs is higher than the percolation threshold, the nanocomposite exhibits a transition of the AC conductivity passing from a capacitive-like (frequency dependent) to a resistive-like (frequency independent) behavior, at a certain cut-off frequency, called the characteristic frequency (*f*_c_). The frequency-independent plateau in the AC conductivity can be found up to 70 Hz (*f*_c_) for the 5 wt%-MWCNT nanocomposite. This value shifts to higher frequencies by increasing the MWCNT content. The overall behavior of *σ_AC_* can be described as the superposition of the capacitive and resistive response, the latter being visible only when the possibility for electrons to pass through the MWCNT conductive network is not negligible. This aspect has been already deeply investigated by Monti et al. [29], who showed similar results related to thermosetting MWCNT-based nanocomposites.

Figure 2b shows the phase angle as a function of frequency of the studied materials. The phase angle is negative without any significant variation at MWCNT contents below the percolation threshold. Over the percolation threshold, the phase angle shows a peak that shifts to higher frequency with increasing MWCNT content. At the highest MWCNT contents, the phase angle tends to zero in the low frequency region, partially hiding the aforementioned peak, corresponding to the current flowing almost exclusively through the conductive MWCNT network, which behaves as a resistive path. This is perfectly coherent with the physical theory, which describes the ohmic behavior of conductors with no phase angle between the electric impulse and the circuit response.

#### 3.1.2. Effect of the Processing Condition 

**Figure 3:**
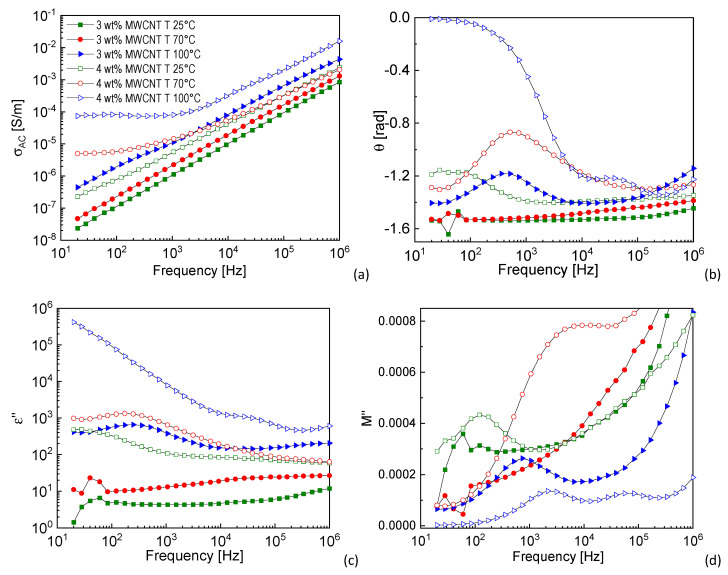
(**a**) *σ_AC_*, (**b**) phase angle, (**c**) *ε”* and (**d**) *M”* of 3 and 4 wt%-multi-walled carbon nanotube (MWCNT) nanocomposites manufactured at temperature of the mold 25-70-100 °C (tests executed in P1; graph legend reported in (**a**) is valid for all the graphs).

Figure 3 shows the dielectric results of the 3 and 4 wt% MWCNT-based nanocomposites manufactured at the three different mold temperature (25, 70 and 100 °C). These contents were selected since they are in the lower part of the range of the electrical percolation threshold. As it is possible to observe, the increase of mold temperature leads to a shift of the dielectric behavior from a fully capacitive to an increasingly higher ohmic behavior. Indeed, from Figure 3a, it can be observed that *σ_AC_* of the sample manufactured with standard conditions (already reported in Figure 2a) has the typical insulating behavior, with the conductivity continuously increasing with the frequency. The conduction mechanism can be ascribed to the tunneling effect rather than the direct contact among the MWCNTs. However, in the case of 4 wt% MWCNT content, with the increase of mold temperature, conductivity becomes frequency-independent in the low frequency region, indicating that a three-dimensional conductive network has been formed and the transport of electrons mainly occurs through it.

The phase angle (Figure 3b) shows a peak at about 400 Hz in the 3 wt%-MWCNT T-100 °C nanocomposite, which cannot be seen at mold temperatures 25 °C and 70 °C. In the 4 wt%-MWCNT samples, the peak is visible at all mold temperatures and it shifts to higher frequency with increasing mold temperature. The same results can be observed in the *ε*” curves reported in Figure 3c, with the peak at about 250 Hz in the 3 wt%-MWCNT T-100 °C nanocomposite and at about 40, 250 and 3800 Hz in the 4 wt%-MWCNT T-25 °C, T-70 °C and T-100 °C nanocomposite, respectively. The presence of a peak in the phase angle and imaginary part of permittivity curves can be associated to a dipole relaxation, whose characteristic time can be evaluated as the opposite of the frequency. In the 3 wt%-MWCNT samples, this peak is visible only at mold temperature 100 °C. This can be associated to a reorganization of the MWCNTs in the PP polymer matrix, induced by this processing condition, which allows the creation of a stronger interaction (detectable from this peak) between the insulating polymer and the MWCNTs [31]. For inhomogeneous materials, as in the case of PP-MWCNT nanocomposite, this relaxation can be attributed to Maxwell–Wagner–Sillars (MWS) interfacial polarization [32,33]. In the case of 4 wt%-MWCNT samples, the shift of the peak to higher frequency means that the relaxation mechanism is faster (lower relaxation time) as the mold temperature increases and that the electric dipoles associated to the interfacial polarization have a higher mobility. In the 4 wt%-MWCNT T-100 °C sample, phase angle curve tends to zero in the low frequency region, which corresponds to the current flowing almost entirely through the MWCNT network. Indeed, this conductive structure behaves as a resistive path. In the imaginary part of permittivity curve, this conductive component masks the interfacial polarization, as it well described in literature [34,35,36].

In order to make it clearer and to overcome this difficulty in evaluating the interfacial polarization, McCrum et al. [37] introduced the electric modulus formalism (M^*^), which is the inverse of complex dielectric permittivity and is defined as $M^{*}=\frac{1}{\varepsilon^{*}}=\frac{\varepsilon^{\prime }}{{\varepsilon^{\prime }}^{2}+{\varepsilon^{\prime \prime }}^{2}}+i\frac{\varepsilon^{\prime \prime }}{{\varepsilon^{\prime }}^{2}+{\varepsilon^{\prime \prime }}^{2}}=M^{\prime }+iM^{\prime \prime }$. M^*^ describes the dynamic characteristics of the charge motion in conductors with regard to relaxation in an electric field [38,39,40]. The use of electric modulus has remarkable advantages in the analysis of the bulk relaxation processes compared to other electrical functions, since by its definition, suppresses undesirable electrode polarization effects that might mask any other response stemming from the interior of the specimen. Recent examples of overcoming huge electrode polarization masking are given in [41,42,43].

Figure 3d shows *M*” as a function of frequency. This graph allows a more in-depth analysis of the dielectric results and represents a further compelling evidence of the presence of a MWS interfacial polarization in the polymer/tube/polymer structure, which is faster as the mold temperature increases. In particular, a barely visible peak is present in the 3 wt%-MWCNT T-25 °C and T-70 °C nanocomposites, while it was not detected in *ε*” and *θ* curves. Since it appears at about 60 and 100 Hz (while the one related to 3 wt%-MWCNT T-100 °C nanocomposite is visible at 740 Hz), this further confirms the tendency of interfacial polarization of being faster as the mold temperature increases. It is worth noting that the *M*” curve of the 4 wt%-MWCNT T-100 °C nanocomposite shows two distinctive peaks, which could be associated to two different tube-polymer interphases.

#### 3.1.3. Position over the Sample 

It is well known that injection molding induces an inhomogeneity in the morphology of the produced component. This is due to the shear stress induced on the molten material, by the flow during the mold-filling phase, which changes over the thickness and the surface of the mold cavity and the produced component [12]. In the case of polymers filled with conductive particles, this inhomogeneity in the morphology, results in an equivalent inhomogeneity in the electrical behavior [3,14,30,41]. As an example, Cesano et al. [30] highlighted this effect in the thickness of PP-MWCNT nanocomposites, while Pötschke et al. [44] studied this phenomenon on several positions over the surface of polycarbonate-MWCNT nanocomposites.

**Figure 4:**
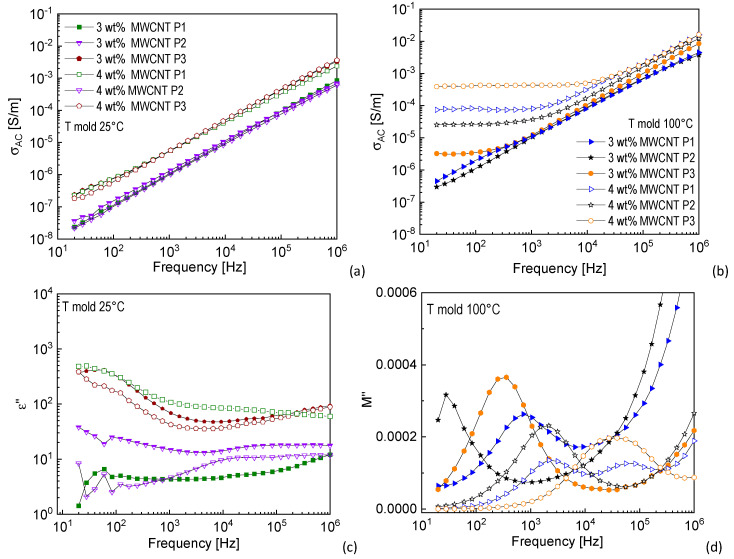
(**a,b**) *σ_AC_* curves of 3 and 4 wt%-MWCNT nanocomposites manufactured at mold temperatures (**a**) 25 °C and (**b**) 100 °C. (**c**,**d**) *M”* curves of 3 and 4 wt%-MWCNT nanocomposites manufactured at mold temperatures (**c**) 25 °C and (**d**) 100 °C (graph legend reported in (**a**) is valid also for (**c**); graph legend reported in (**b**) is valid also for (**d**)).

Figure 4 reports the results of the dielectric properties of 3 and 4 wt%-MWCNT nanocomposites, manufactured at mold temperatures 25 and 100 °C, as measured in the studied different positions over the surface of the sample. As it is possible to observe from Figure 4a,b, AC electrical conductivity of the samples manufactured at mold temperature 25 °C, regardless of the MWCNT content and the tested position, shows the typical insulating trend, with the conductivity continuously increasing with frequency. As for the results obtained with mold temperature 100 °C, the 3 wt%-MWCNT sample shows a plateau in the low frequency region in P3, which means that, in this position, at least a weak conductive MWCNT path is formed. The 4 wt%-MWCNT sample shows in all the positions a plateau in the low frequency region, with a clear tendency of increasing conductivity when moving from the position closest to the gate to the position further from the gate. Therefore, it seems that conductivity in P3 is always higher than in the other positions, whereas P2 represents in all cases the least conductive zone. P3 represents the area where the flux of the molten material reaches the end of the cavity. Therefore, the shear stress in this region is most likely lower if compared with all the other positions, because the velocity of the flux is rapidly going to zero. As a consequence, it turns out that this lower shear stress leads to a rearrangement of the MWCNT clusters and a PP crystalline structure, which is more efficient in terms of electrical conduction mechanism.

The *ε*” and *M*” curves reported in Figure 4c,d are related to the samples manufactured at mold temperatures 25 °C and 100 °C, respectively. They show the presence of a peak related to the interfacial polarization, which in the case of mold temperature 100 °C (Figure 4d) tends to shift to higher frequency, as the tested position moves from the gate to the end of the cavity. This further confirms that when the conductive mechanism is more efficient, the MWS interfacial relaxation is faster.

### 3.2. Crystalline Structure and MWCNT Cluster Morphology

The relationship of the processing condition and tested position over the surface of the sample, with the morphology have been investigated following two different approaches, the former focused on the crystalline structure, the latter to the morphology of the MWCNT clusters embedded in the polymer matrix. XRD was performed in order to thoroughly understand how the injection molding process affects the crystalline structure of the produced samples, with the final aim of correlating it with a diverse MWCNT percolation network and dielectric behavior measured and observed. As reported in literature, PP shows three polymorphic crystallographic forms: monoclinic *α*-phase, a hexagonal *β*-phase and a triclinic *γ*-phase [45]. Each crystalline form presents its own characteristic peaks in the XRD pattern. In the reported XRD graph, the following peaks at *2θ* = 14.0°, 16.9°, 18.7°, 21.2°, 21.8° and 25.4° are related to the (110), (040), (120), (131), (041) and (060) crystalline planes of α-PP, respectively. The triclinic *γ*-phase corresponds to *2θ* = 20.1°, which is associated to the (117) crystalline plane [21,45]. The *γ*-phase fraction (*X_γ_*) can be estimated by the formula $X_{\gamma }=\frac{h_{\gamma }}{h_{\gamma }+h_{\alpha }}$, where *h_γ_* and *h_α_* are the peak height of the two crystallographic planes (117) and (120), representative of the two phases [21,46,47]. The results related to the 3 wt%-MWCNT samples are reported in Table 1.

**Figure 5:**
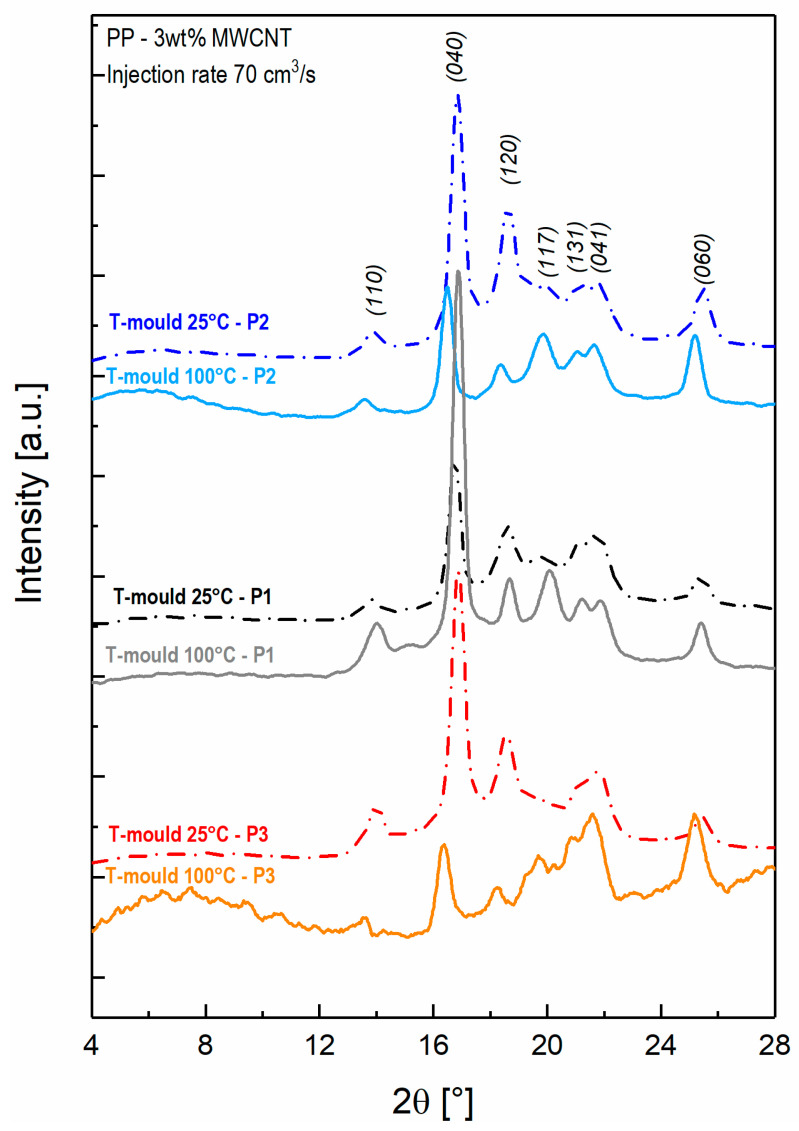
X-ray diffraction patterns of 3 wt% MWCNT nanocomposites, manufactured at mold temperatures 25 °C and 100 °C in the three positions over the samples.

Figure 5 reports the XRD spectra of 3 wt% MWCNT nanocomposites produced at 25 °C and 100 °C mold temperature, as measured in the three different positions. As it is possible to observe, an increase of mold temperature results in an increase of intensity of (117) peak, indicating that *γ*-phase is favored at slower cooling rate (achieved when higher mold temperatures are applied) [47]. This tendency is obtained in all the studied positions and further confirmed by the results reported in Table 1. This outcome, together with the dielectric results, which show an increase of the electrical conductivity and faster interfacial polarization mechanism at a slower cooling rate, demonstrates that PP *γ-*phase leads to the formation of a more efficient MWCNT conductive system.

**Figure 6:**
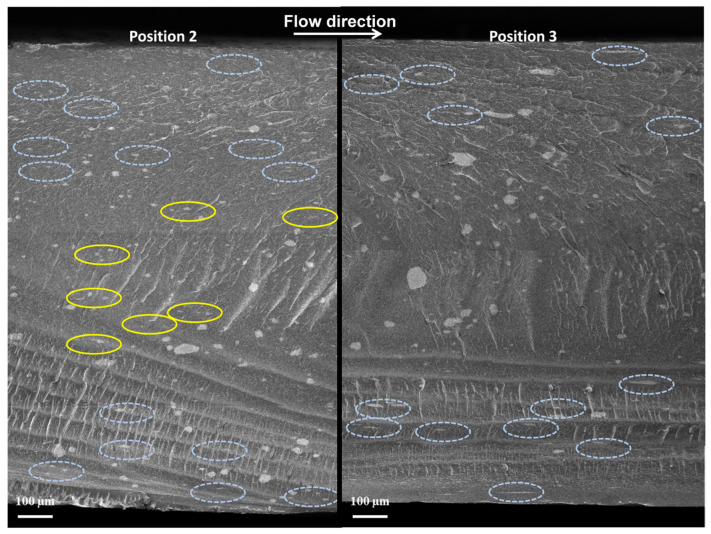
FESEM images of the cross section of 3 wt%-MWCNT T-100 °C samples in P2 and P3.

Figure 6 shows the FESEM images of the global cross section of the 3 wt%-MWCNT T-100 °C sample, in P2 and P3. Similar results have been obtained at mold temperature 25 °C (not reported in this paper). In both cases, it can observe that MWCNT clusters with different dimension and shape are evenly distributed in the whole section of the samples. More in detail, as it is possible to observe, the MWCNT clusters are more elongated in the flux direction in P2, even in the core region. On the other hand, P3 is characterized by the presence of clusters with a more homogeneous shape, especially in the core region.

**Figure 7:**
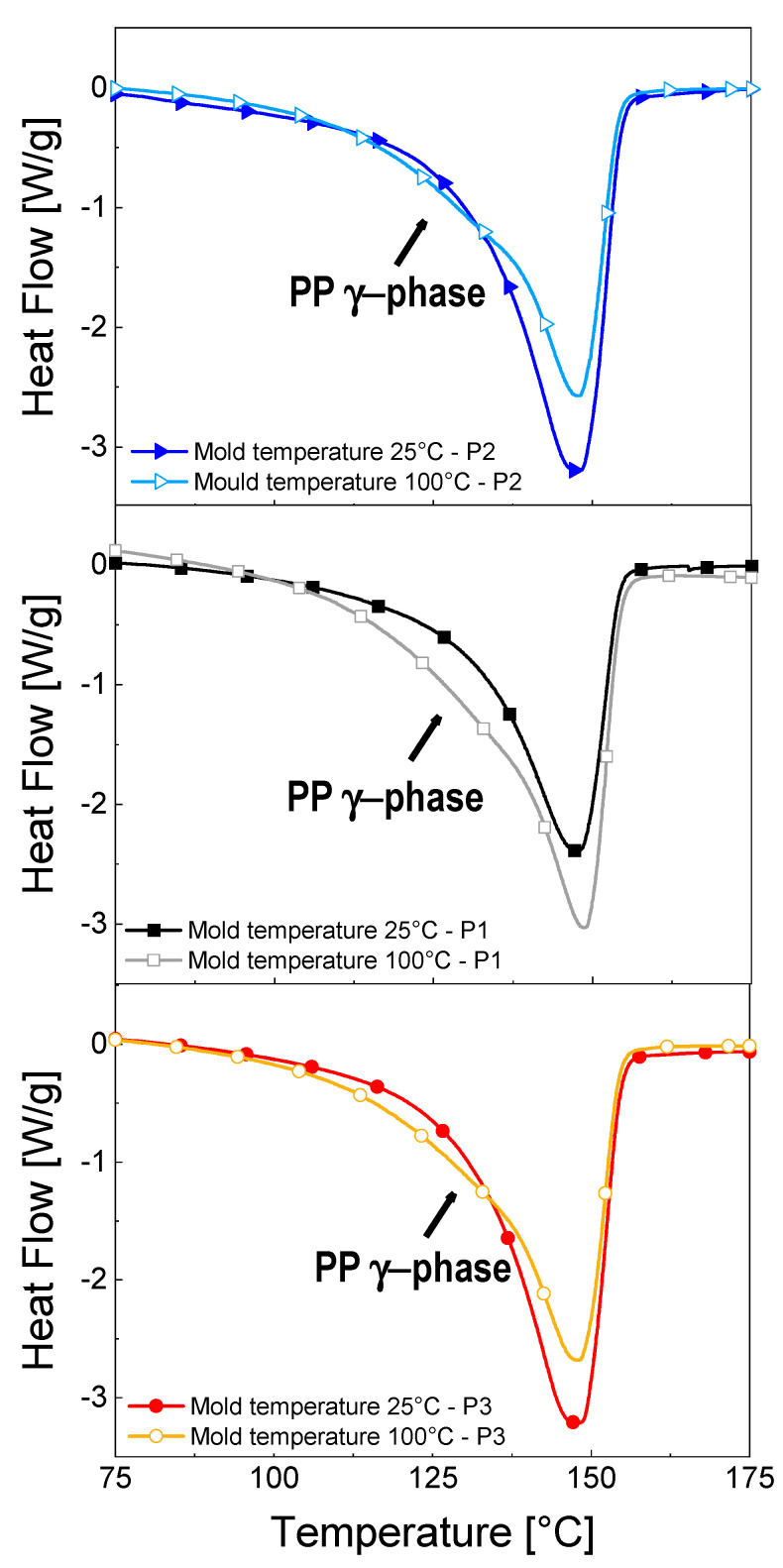
DSC thermograms of 3 wt%-MWCNT nanocomposites manufactured at 25 °C and 100 °C, in the different positions (exo up).

Finally, Figure 7 and Table 2 report the DSC curves and the degree of crystallinity of 3 wt%-MWCNT nanocomposites, manufactured with the mold temperature set at 25 and 100 °C, in the three studied positions. Observing results reported in Table 2, it is possible to notice that the sample manufactured with mold temperature 100 °C shows a higher crystallinity if compared to the sample manufactured with mold temperature 25 °C, with no significant difference related to the position over the surface of the sample. Moreover, as it can be observed from the thermograms of Figure 7, a broad melting peak, formed by a second peak in the temperature range between 120 °C and 145 °C, partially hidden by the principal peak (at around 150 °C), is observable for the nanocomposites produced with a mold temperature of 100 °C. This second peak was ascribed to the melting of the PP *γ*-crystals by Zhu et al. [21]. Hence, the increasing broadness of this peak with the increment of mold temperature is consistent with the obtained results for the XRD estimations, where an increase of *X_γ_* in the 3 wt%-MWCNT occurs when identical processing condition are applied.

## 4. Conclusions

This paper reports a study on the correlation between the dielectric properties of injection-molded PP-MWCNT nanocomposites and the related morphology, focusing on how they are influenced by tuning the mold temperature of the injection molding process. The produced crystalline structure has been investigated through X-ray diffraction and DSC, while MWCNT cluster morphology has been observed by FESEM microscopy.

The AC electrical conductivity of MWCNT-based nanocomposites shows less dependency on frequency in the low-frequency region and at high MWCNT content, which is typically associated to a shift from a fully capacitive to a resistive behavior. This phenomenon was also confirmed by the phase angle curves, which tends to zero with increasing MWCNT content in the low frequency region. The effect of the temperature of the mold was studied on 3 and 4 wt% MWCNT nanocomposites and has shown a change of the dielectric behavior from a fully capacitive to a partially resistive behavior, as mold temperature increases. This indicates the formation of an MWCNT network, which allows the transport of electrons. Moreover, as the conductive network becomes more efficient, a peak appears in the phase angle and in the imaginary part of permittivity curves. This peak can be associated to the Maxwell–Wagner–Sillars (MWS) interfacial polarization. The analysis described so far has been performed in the center of the sample. Test were also performed in other two positions over the surface of the samples, one closer to the gate (P2), one close to the end of the cavity (P3). The dielectric results of the samples manufactured at mold temperature 100 °C demonstrate that P3 is the most conductive, whereas the P2 represents the least conductive zone.

The morphological study performed by XRD and DSC has shown that an increase of mold temperature results in an increase of PP *γ*-phase crystalline structure. The correlation with the dielectric results indicates that this crystalline structure favors the formation of a more efficient MWCNT conductive network. The effect of the position over the surface of the sample on the morphology has been studied also by FESEM microscopy. As a result, P3 is characterized by the presence of clusters with a more homogeneous shape, especially in the core region, which can be most likely associated to the lower shear stress on the molten material in this region of the cavity, if compared with all the other regions, due to the completion of the filling phase.

In conclusion, this paper has shown that tuning the injection molding processing conditions can drastically modify the frequency-dependent electrical behavior when the MWCNT content is in the range of the electrical percolation threshold. This has been associated to the selective formation of PP *γ*-phase crystalline structure, in the presence of higher temperature of the mold. The achieved results enlarge the awareness of the significance of the advanced process design of electrically conductive injection-molded components. The in-depth understanding of the possibility in tuning the frequency-dependent electrical behavior, and how it can be measured by XRD, can offer an essential step forward for the delineation of optimized processing procedures.

## Figures and Tables

**Table 1:** Fraction of *γ*-phase in 3 wt% MWCNT nanocomposite manufactured at mold temperature 25–100 °C and in the different positions over the samples.

Amount of *γ-*Phase			
Mold Temperature	Positions over the Sample		
	2	1	3
**25 °C**	7%	14%	45%
**100 °C**	63%	54%	55%

**Table 2:** Degree of crystallinity of 3 wt%-MWCNT nanocomposites as a function of mold temperature in the different positions over the sample.

Degree of Crystallinity, *X*_c_ (%)			
Mold Temperature	Positions over the Sample		
	2	1	3
**25 °C**	34.6	35.1	34.9
**100 °C**	38.5	39.4	38.7

## Data Availability

The data presented in this study are available on request from the corresponding authors.
